# Case Report: Multisystem inflammatory syndrome in children with associated proximal tubular injury

**DOI:** 10.3389/fneph.2023.1194989

**Published:** 2023-06-19

**Authors:** Silvia Maria Orsi, Carlotta Pepino, Lisa Rossoni, Margherita Serafino, Roberta Caorsi, Stefano Volpi, Serena Palmeri, Alessandro Faragli, Francesca Lugani, Carolina Bigatti, Gian Marco Ghiggeri, Enrico Eugenio Verrina, Edoardo La Porta, Andrea Angeletti

**Affiliations:** ^1^ Department of Neuroscience, Rehabilitation, Ophthalmology, Genetics, Maternal and Child Health, University of Genoa, Genoa, Italy; ^2^ Department of Pediatric Cardiology and Cardiac Surgery, IRCCS Istituto Giannina Gaslini, Genoa, Italy; ^3^ Center for Autoinflammatory Diseases and Immunodeficiencies, IRCCS Istituto Giannina Gaslini, Genoa, Italy; ^4^ Department of Internal Medicine and Cardiology, Charité – Universitätsmedizin Berlin, Berlin, Germany; ^5^ Division of Nephrology, Dialysis and Transplantation, IRCCS Istituto Giannina Gaslini, Genoa, Italy; ^6^ Laboratory of Molecular Nephrology, IRCCS Istituto Giannina Gaslini, Genoa, Italy; ^7^ Dialysis Unit, Department of Pediatric, IRCCS Istituto Giannina Gaslini, Genoa, Italy

**Keywords:** MIS-C multisystem inflammatory syndrome in children, proximal tubule injury, bladder debris, Fanconi syndrome, kidney injury, SARS – CoV – 2

## Abstract

**Introduction:**

SARS-CoV-2 infection in the pediatric population can be associated with a multiorgan inflammatory syndrome called children’s multisystem inflammatory syndrome (MIS-C). The kidneys can be affected by a broad spectrum of possible injuries, whose pathogenetic mechanisms are still unclear.

**Case report**

We report the case of a 5-year-old boy with severe cardiac involvement in the context of MIS-C. After two weeks of hospitalization, an abdominal ultrasound showed massive bladder “debris”, followed by the onset of normoglycemic glycosuria. Over time, there was a progressive increase in glycosuria, and the presence of a mat of amorphous phosphate crystals was evidenced on urinary sediment. Together with the findings of hypo-uricemia, increased urinary uric acid, and globally increased urinary amino acids, a clinical picture of kidney proximal tubular damage with secondary Fanconi-like syndrome took shape.

**Discussion:**

This case report describes the case of a patient with MIS-C with cardiac and kidney involvement characterized by proximal tubular damage, which slowly improved but still persisted at the 8-month follow-up. The pathogenesis of the damage is unclear and probably multifactorial.

## Introduction

Kidney dysfunction is a common consequence of SARS-CoV-2 infection (1, 2), having been reported in adults and, to a lesser degree, in children. Kidney consequences of COVID-19 can include a broad spectrum of damages, ranging from acute kidney injury (AKI) with glomerular or tubular injury to mild proteinuria and/or hematuria (3).

In children, SARS-CoV-2 infection can manifest as a multisystem inflammatory syndrome (MIS-C) that typically occurs 3–6 weeks after mild or asymptomatic COVID-19 disease (4). This rare disorder is characterized by a hyperinflammatory state with a range of clinical presentations that can involve multiple organs with a generalized increase in inflammatory biomarkers, such as C-reactive protein (CRP), ferritin, D-dimer, and lactate dehydrogenase (5, 6). Common manifestations include fever, rash, abdominal pain, and gastrointestinal symptoms, mimicking appendicitis in some children (4). Cardiological involvement is characterized by diminished left ventricular systolic function with or without coronary artery abnormalities, fluid overload or hypotension, and an increase in pro-brain natriuretic peptide and cardiac enzymes. The kidneys can be affected by a broad spectrum of possible injuries; the incidence of AKI ranges from 10% to 46% (7) and its pathogenetic mechanisms are still unclear and probably multifactorial (8, 9). 

Here, we report a case of MIS-C with peculiar nephro-urological involvement and ultra-sonographic features characterized by proximal tubule dysfunction.

## Case description

**Figure 1:**
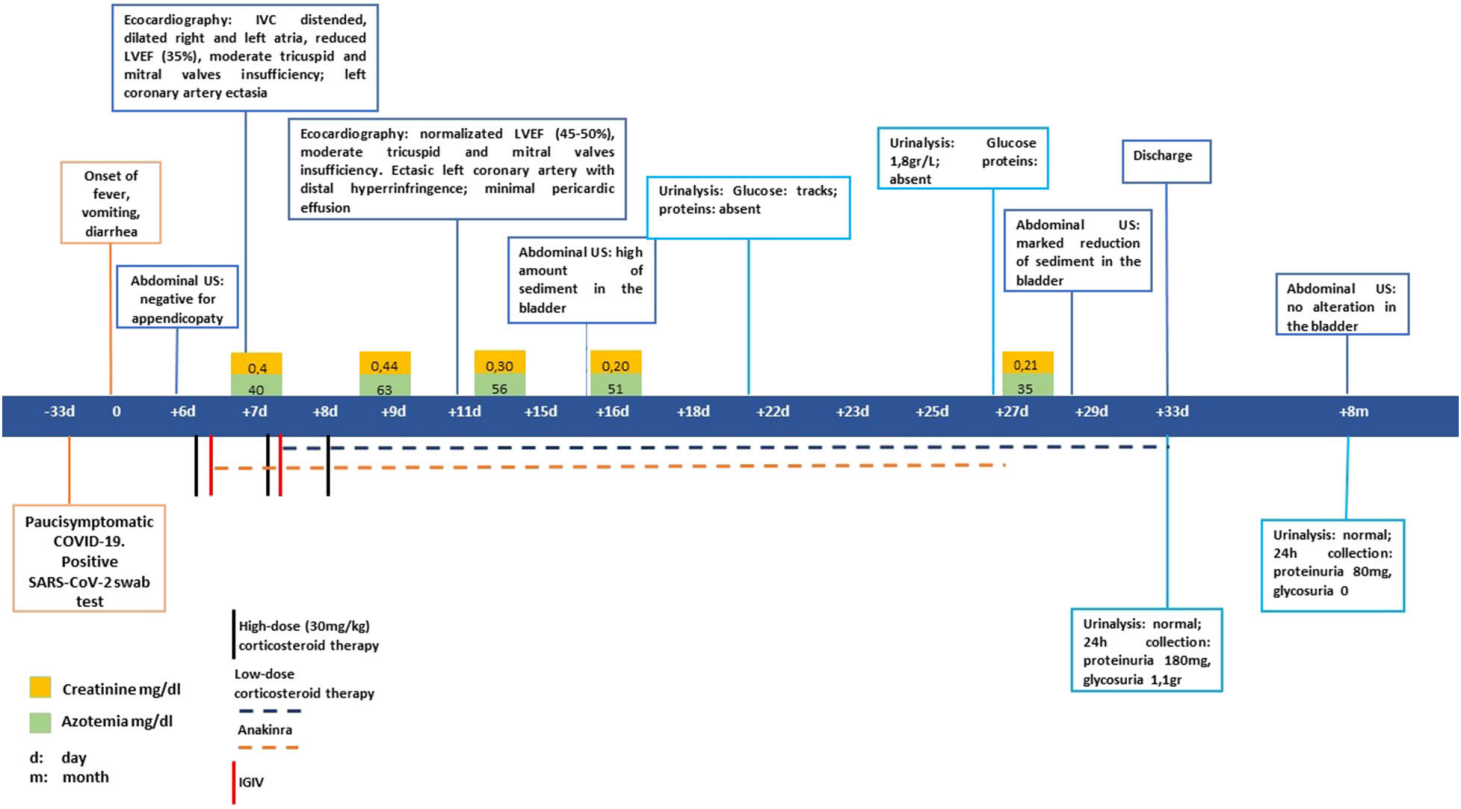
Timeline of clinical events, diagnostic examinations, and treatments of the patient.

A previously healthy 5-year-old boy was admitted to hospital in February 2022 due to fever lasting for 6 days with spikes up to 40°C, accompanied by vomiting, diarrhea, and intense abdominal pain. Because of suspected intestinal adenitis with neutrophilic leukocytosis and markedly increased CRP (26 mg/dL n.l. < 0.46), the patient underwent a laparoscopic appendicectomy that showed an uninjured appendix. Considering the recent paucisymptomatic SARS-CoV-2 infection confirmed by an antigenic pharyngo-nasal swab test and the persistent fever unresponsive to antibiotics, further investigations were carried out. Blood tests revealed the presence of a hypochloremic metabolic alkalosis and increased blood urea nitrogen (max value 60mg/dl), troponin I, and NT-Pro-BNP, while other parameters were normal, including urine examination (see **Table 1**). On admission, Creatinine was 0.4 mg/dL. It then progressively decreased and stabilized at 0.2-0.25 mg/dL, together with the decrease in blood urea nitrogen values. The echocardiogram demonstrated diffuse hypokinesia with a reduced left ventricular ejection fraction (LVEF 35%), a right ventricle with volumetric overload, and a left coronary artery ectasia. The case was suggestive of MIS-C; therefore, steroids (methylprednisolone 30mg/kg), immunoglobulins (IGIV 1gr/kg), and immunosuppressive therapy with anti-IL-1 receptor antagonist (Anakinra 200mg twice daily) were started. In addition, supportive therapy with furosemide was administered and, on day 14, an ACE inhibitor drug (enalapril 0.05mg/kg) was added due to persisting high values of arterial blood pressure. Finally, anticoagulant therapy with heparin (2000 IU/day) was started and was later replaced by anti-platelet therapy (aspirin 75 mg/day) on day 11 (see **Figure 1**).

**Table 1:** Patient’s blood and urine tests data.

	Admission	+2 days from admission	+14 days from admission	+22 days from admission	+33 days from admission	8 months follow-up
**Hemoglobine (g/dl)**	10,5	10	12,6	–	12	12,4
**White blood cells (n°/uL)**	24350	12900	24030	–	12300	11200
** Neutrophils (n°/uL)**	21570	9040	13070	–	8040	4090
** Lymphocytes (n°/uL)**	1660	2200	9060	–	3090	5800
**Platelets (n°/uL)**	429000	500000	580000	–	324000	512000
**Creatinine (mg/dL)**	0,4	0,44	0,25	–	0,2	0,3
**Blood urea nitrogen (mg/dL)**	40	60	36	–	35	46
**GOT (U/L)**	40	30	40	–	30	27
**GPT (U/L)**	60	50	80	–	50	19
**CRP (mg/dL) [n.l. < 0.46)]**	17,87	6	neg	–	neg	neg
**Procalcitonin (ng/ml) [n.l. < 0.5)]**	48,87	15	–	–	–	–
**Ferritin (ng/ml)**	1277	428	968	–	–	15
**NT-pro BNP (pgr/ml)**	> 35000	13286	182	–	–	17
**Troponin I (ng/ml) [n.l. <10]**	neg	0,17	–	–	neg	neg
**Albumin (mg/dl)**	2400	3500	3664	–	3370	4065
**D-dimer (mg/L FEU) [n.l. < 0.55)]**	12,37	5,45	0,62	–	neg	neg
**Uric acid (mg/dl) [n.l. > 2]**	–	2,7	2,5	–	1,6	3,7
**Proteinuria on 24h collection (g/24h) [n.l. < 0.15]**	–	–	–	–	0,18	0,08
**Glycosuria on 24h collection (g/24h) [n.l < 0.1]**	–	–	–	–	1,1	0
**Natriuresis on 24h collection** **(mEq/24h) [n.l < 150]**	–	–	–	–	231,4	171
**Chloruria on 24h collection (mEq/24h) [n.l < 125]**	–	–	–	–	247,7	167,8
**Uricuria (mg/dl)**	–	–	–	–	170,2	114,4
**Phosphaturia (mg/dl)**	–	–	–	–	184,2	95,9
**rCaU/CrU [n.l < 0.2]**		–	–	–	0,66	0,15
**Urinalysis**	–	–	Glucose: tracks; proteins: absent	Glucose 1,8gr/L; proteins: absent	Glucose and proteins absent	Glucose and proteins absent
Urinary amino acids chromatography (umol/mmcrea)						
ALA [n.l 27 – 92]	–	–	–	–	281	178
ARG [n.l 0 - 7]	–	–	–	–	21	20
ASP [n.l 2 - 8]	–	–	–	–	47	–
CYS [n.l 4 - 11]	–	–	–	–	25	10
GLN [n.l 52 - 133]	–	–	–	–	486	210
GLY [n.l 91 - 246]	–	–	–	–	800	619
HIS [n.l 61 - 216]	–	–	–	–	729	293
LYS [n.l 10 - 68]	–	–	–	–	379	285
ORN [n.l 0 - 7]	–	–	–	–	8	6
SEU [n.l 38 - 93]	–	–	–	–	587	322
TAU [n.l 17 - 230]	–	–	–	–	278	285
THR [n.l 9 - 39]	–	–	–	–	518	257

GOT, Glutamic Oxaloacetic Transaminase; GPT, Glutamate Pyruvate Transaminase; CRP, C-Reactive Protein.

The therapy was well tolerated, and progressive clinical, laboratory, and instrumental improvements were observed. In particular, the echocardiograms showed a progressive normalization of LVEF and the left coronary artery (**Figure 1**).

**Figure 2:**
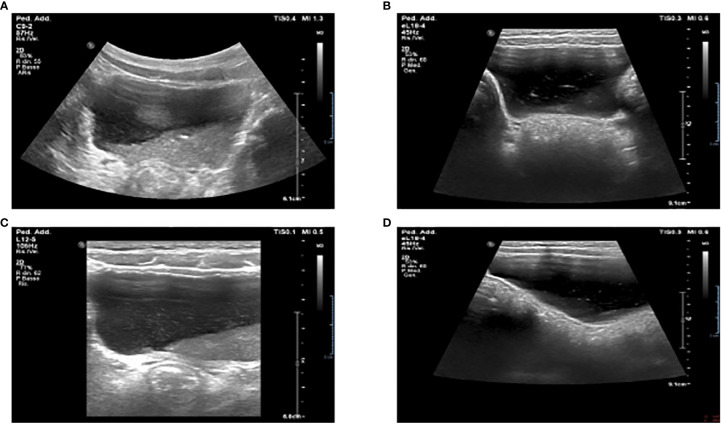
Ultrasound examination showing massive bladder debris in a 5-year-old child affected by MIS-C **(A, B)**. First incidental finding of massive sediment and floating bladder debris at abdominal ultrasound examination. **(C, D)**. Reduction of the debris after X days at ultrasound control examination.

On day 18, during an abdominal ultrasound performed as surgery follow-up, the presence of bladder debris was discovered (**Figures 2A, B**), despite the absence of other signs of urinary tract infection.

Thus, more examinations were performed. They showed a persistent normal renal function with normoglycemic glycosuria, confirmed by the urinary sediment and the 24-hour urine collection (glucose 1.1 g/24h n.l < 0.1), together with hypercalciuria (rCaU/CrU 0.66 n.l < 0.2) and mild proteinuria (0.18 g/24h n.l. < 0.15). In addition, the urinary sediment reported numerous amorphous phosphate crystals. Due to suspicions of Fanconi syndrome, further tests were performed: uricemia had decreased (1,6 mg/dl n.l. > 2) and hyperuricosuria was found together with a significant increase in all urinary amino acids excreted (see **Table 1**). During the ultrasound examination performed on day 29, a sharp decrease of multiple echoes of hyperechogenic corpuscular material in suspension was reported, with no morphological abnormalities of the kidneys and urinary tract (**Figures 2C, D**).

Thus, after a few days, the patient was discharged in good condition, with normal echocardiography and urinary exams. They were given instructions to continue with oral steroid therapy and antiplatelet and antihypertensive therapy, which were later stopped due to the normalization of blood pressure values. At the 8-month follow-up, no bladder debris was present upon ultrasound examination, while urinalysis showed an improvement of persistent proximal renal tubule damage characterized by increased amino acids, sodium chloride, and uric acid excretion.

## Discussion

We have described the case of a child diagnosed with MIS-C with severe cardiac involvement, who experienced alterations in urinalysis consisting of normo-glycemic glycosuria, amino-aciduria, hyperuricosuria, and the presence of amorphous phosphate crystals. These findings, together with decreased uricemia, are consistent with proximal tubule damage, more precisely, a Fanconi-like syndrome tubulopathy that persists over months. MIS-C is thought to be an exaggerated immune response to SARS-CoV-2 infection, but the exact pathogenesis of multiorgan dysfunction is still unknown. Very few studies have described the incidence and characteristics of renal complications in MIS-C. AKI is frequently reported in children diagnosed with this disorder (7–10), but no studies have specifically reported acute tubular involvement.

The pathophysiology of renal dysfunction seems to be multifactorial. Hemodynamic, iatrogenic, viral, or immune-mediated causes could have all contributed to the development of both pre-renal and renal parenchymal effects (7). Firstly, the reduced cardiac ejection fraction and the capillary leakage due to the inflammatory state both lead to kidney hypoperfusion and consequent ischemic damage, ischemic tubular damage. Secondly, our patient experienced a subclinical AKI, with increased values of blood urea nitrogen; creatinine, albeit not enough to meet the KDIGO (Kidney Disease Improving Global Outcomes) criteria for AKI; and elevated serum urea/creatinine ratio. Moreover, the contribution of drug toxicity to kidney injury cannot be excluded. Diuretics and iACE could have also contributed to glomerular hypoperfusion and consequent pre-renal damage. However, iatrogenic kidney damage due to steroids, IL-1 receptor antagonists, or immunoglobulins is very unlikely and can be excluded. Finally, a possible contribution of immune overactivation or a direct kidney tropism of the SARS-CoV-2 virus leading to tubular injury and podocytopathy cannot be ruled out. SARS-CoV-2 virus is suggested to reach proximal tubule and podocytes through spike (S) glycoprotein and ACE-2 receptor binding, and the consequent transmembrane serine proteases (TMPRSSs) action, which facilitates membrane fusion (7, 11–13). In the human kidney, ACE-2 and TMPRSSs are expressed in the nephron and demonstrate high tropism, primarily in the proximal tubule apical membrane, along with other proteases necessary for SARS-CoV-2 (14, 15). The development of a hyperinflammatory state with similar aspects to cytokine release syndrome has been hypothesized, with a possible crucial role of IL-6 IL-2R (7) in worsening renal function.

Proximal tubule dysfunction in adults was investigated in a recent study by A. Werion et al. (12). They showed that the dysfunction occurs early during the course of SARS-CoV-2 infection, and it is characterized by low molecular weight proteinuria, defective handling of uric acid and phosphate, and aminoaciduria. Normoglycemic glycosuria was not evidenced in their cohort of patients. Moreover, the aminoaciduria they detected in 46% of patients tested was limited to neutral amino acids, while in our patient, a generalized aminoaciduria was found.

Our patient did not present a clinical AKI, but the results obtained are coherent with an alteration of the proximal renal tubular structure. Because we chose not to perform a kidney biopsy, the exact etiology of the damage is unknown, and we can only speculate on the possible causes behind it. The abovementioned presence of bladder debris could be associated with a concomitant urinary tract infection (16), a clinical condition that was ruled out in our patient. Nevertheless, the significance of bladder debris in the alteration of urine analysis is still unclear and deserves further study (17). In our case, we ruled out the presence of urinary tract infection. Thus, the significance of bladder debris is attributable to an aspecific urine alteration with a massive presence of phosphate crystals.

Subclinical AKI, defined as the presence of kidney dysfunction not meeting the criteria for AKI, has been described in SARS-CoV-2 infection. Even if clinical data are still poorly known, especially in MIS-C, it seems to be correlated to a more severe course of the disease (18). Our patient experienced a subclinical AKI by presenting, at admission, serum creatinine values that were double those when discharged. These persisted for 10 days before starting to decrease. A few days after reaching the peak of serum creatine, abdominal US was performed with the incidental evidence of bladder debris and urinary alterations. Unfortunately, no urine examinations were performed before this because there was no suspicion of nephron-urological involvement. We, therefore, cannot determine exactly when kidney tubular injury arose.

Increasing evidence suggests that subclinical AKI and urinary alterations are clinically significant and independently associated with adverse outcomes (19, 20). It also underlines the important role of urinary biomarkers and urinary analysis in the recognition of precocious subclinical and clinical AKI (21, 22).

Regardless of the etiopathology of the tubular injury, this illustrative case aims to emphasize the relevance of urinary examinations in this clinical setting, with the ultimate goal of aiding the early recognition of clinical and subclinical AKI.

The primary goals of therapy in MIS-C are to reduce systemic inflammation, to give hemodynamic support in cases of cardiac dysfunction, and to treat singular organ involvement. Most widely used pharmacologic approaches consider IVIG and steroids, anakinra (IL-R1 antagonist), infliximab (TNF-alfa blocker), or tocilizumab (IL-6 antagonist) in cases of persistent inflammatory state and poor response to first-line therapy (23). If renal damage is present, the therapy is based, at first, on renal supportive care, optimizing hemodynamics through infusive therapy or diuretics, depending on the volemic status of the patient. Critical cases could require kidney replacement therapy (7).

In conclusion, we suggest considering possible renal involvement in cases of MIS-C, and in particular, assessing renal function and performing frequent urine tests in order to recognize AKI and dysfunction of the kidney proximal tubule as early as possible.

## Data availability statement

The original contributions presented in the study are included in the article/supplementary material. Further inquiries can be directed to the corresponding author.

## Ethics statement

Written informed consent was obtained from the minor(s)’ legal guardian/next of kin for the publication of any potentially identifiable images or data included in this article.

## Author contributions

SO and EP contributed to the conception of the study and wrote the paper. MS, AA, GMG, CP, SV, SP, AF, FL, CB and EV reviewed the manuscript and contributed to the final draft. All authors contributed to the article and approved the submitted version.

## References

[1] La Porta E Baiardi P Fassina L Faragli A Perna S Tovagliari F. The role of kidney dysfunction in COVID-19 and the influence of age. Sci Rep (2022) 12(1):8650. doi: 10.1038/s41598-022-12652-0 PMC9125966 35606394

[2] Naicker S Yang CW Hwang SJ Liu BC Chen JH Jha V. The novel coronavirus 2019 epidemic and kidneys. Kidney Int (2020) 97(5):824–8. doi: 10.1016/j.kint.2020.03.001 PMC7133222 32204907

[3] Serafinelli J Mastrangelo A Morello W Cerioni VF Salim A Nebuloni M. Kidney involvement and histological findings in two pediatric COVID-19 patients. Pediatr Nephrol (2021) 36(11):3789–93. doi: 10.1007/s00467-021-05212-7 PMC8371583 34406477

[4] Gottlieb M Bridwell R Ravera J Long B. Multisystem inflammatory syndrome in children with COVID-19. Am J Emergency Med (2021) 49:148–52. doi: 10.1016/j.ajem.2021.05.076 PMC8185530 34116467

[5] Feldstein LR Rose EB Horwitz SM Collins JP Newhams MM Son MBF. Multisystem inflammatory syndrome in US children and adolescents. N Engl J Med (2020) 383:334–46. doi: 10.1056/NEJMoa2021680 PMC7346765 32598831

[6] Centers for disease control and prevention: multisystem inflammatory syndrome (MIS-c): health department–reported cases of multisystem inflammatory syndrome (MIS-c) in the united states (2020). Available at: https://www.cdc.gov/mis/mis-c/hcp_cstecdc/index.html.

[7] Sethi SK Rana A Adnani H McCulloch M Alhasan K Sultana A. Kidney involvement in multisystem inflammatory syndrome in children: a pediatric nephrologist’s perspective. Clin Kidney J (2021) 14(9):2000–11. doi: 10.1093/ckj/sfab073 PMC8083308 34471522

[8] Tripathi AK Pilania RK Bhatt GC Atlani M Kumar A Malik S. Acute kidney injury following multisystem inflammatory syndrome associated with SARS-CoV-2 infection in children: a systematic review and meta-analysis. Pediatr Nephrol (2023) 38(2):357–70. doi: 10.1007/s00467-022-05701-3 PMC9362633 35943577

[9] Grewal MK Gregory MJ Jain A Mohammad D Cashen K Ang JY. Acute kidney injury in pediatric acute SARS-CoV-2 infection and multisystem inflammatory syndrome in children (MIS-c): is there a difference? Front Pediatr (2021) 9:692256. doi: 10.3389/fped.2021.692256 34434905 PMC8380850

[10] Lipton M Mahajan RG Kavanagh C Shen CL Batal I Dogra S. Acute kidney injury in COVID-19-associated multisystem inflammatory syndrome in children (MIS-c). Kidney360 (2021) 2:611–8. doi: 10.34067/KID.0005372020 PMC8791329 35373052

[11] Batlle D Soler MJ Sparks MA Hiremath S South AM Welling PA. COVID-19 and ACE2 in cardiovascular, lung, and kidney working group. acute kidney injury in COVID-19: emerging evidence of a distinct pathophysiology. J Am Soc Nephrol (2020) 31(7):1380–3. doi: 10.1681/ASN.2020040419 PMC7350999 32366514

[12] Werion A Belkhir L Perrot M Schmit G Aydin S Chen Z. SARS-CoV-2 causes a specific dysfunction of the kidney proximal tubule. Kidney Int (2020) 98(5):1296–307. doi: 10.1016/j.kint.2020.07.019 PMC7416689 32791255

[13] Pan XW Xu D Zhang H Zhou W Wang LH Cui XG. Identification of a potential mechanism of acute kidney injury during the COVID-19 outbreak: a study based on single-cell transcriptome analysis. Intensive Care Med (2020) 46:1114–6. doi: 10.1007/s00134-020-06026-1 PMC7106051 32236644

[14] Zou X Chen K Zou J Han P Hao J Han Z. Single-cell RNA-seq data analysis on the receptor ACE2 expression reveals the potential risk of different human organs vulnerable to 2019- nCoV infection. Front Med (2020) 14:185–92. doi: 10.1007/s11684-020-0754-0 PMC7088738 32170560

[15] Rahmani W Chung H Sinha S Bui-Marinos MP Arora R Jaffer A. Attenuation of SARS-CoV-2 infection by losartan in human kidney organoids. IScience (2022) 25(2):103818. doi: 10.1016/j.isci.2022 35106453 PMC8795780

[16] McQuaid JW Kurtz MP Logvinenko T Nelson CP. Bladder debris on renal and bladder ultrasound: a significant predictor of positive urine culture. J Pediatr Urol (2017) 385:e1–385.e5. doi: 10.1016/j.jpurol.2017.04.020 PMC5623625 28595971

[17] Cheng SN Phelps A. Correlating the sonographic finding of echogenic debris in the bladder lumen with urinalysis. J Ultrasound Med (2016) 35(7):1533–40. doi: 10.7863/ultra.15.09024 27246660

[18] Silva-Aguiar RP Teixeira DE Peres RAS Peruchetti DB Gomes CP Schmaier AH. Subclinical acute kidney injury in COVID-19: possible mechanisms and future perspectives. Int J Mol Sci (2022) 23(22):14193. doi: 10.3390/ijms232214193 36430671 PMC9693299

[19] Fang F Hu X Dai X Wang S Bai Z Chen J. Subclinical acute kidney injury is associated with adverse outcomes in critically ill neonates and children. Crit Care (2018) 22:256. doi: 10.1186/s13054-018-2193-8 30305134 PMC6180629

[20] Zou C Wang C Lu L. Advances in the study of subclinical AKI biomarkers. Front Physiol (2022) 13:960059. doi: 10.3389/fphys.2022.960059 36091391 PMC9449362

[21] Perazella MA Cosa S. Urine microscopy is associated with severity and worsening of acute kidney injury in hospitalized patients. Clin J Am Soc Nephrol (2010) 5(3):402–8. doi: 10.2215/CJN.06960909 PMC2827582 20089493

[22] Li N Zhou WJ. Association between urine microscopy and severe acute kidney injury in critically ill patients following non-cardiac surgery: a prospective cohort study. Ann Palliative Med (2022) 22(7):2327–37. doi: 10.21037/apm-21-3085 35610195

[23] Henderson LA Canna SW Friedman KG Gorelik M Lapidus SK Bassiri H. American College of rheumatology clinical guidance for multisystem inflammatory syndrome in children associated with SARS-CoV-2 and hyperinflammation in pediatric COVID-19: version 3. Arthritis Rheumatol (2022) 74(4):e1–e20. doi: 10.1002/art.42062 35118829 PMC9011620

