# Corrigendum: Case report: Multisystem inflammatory syndrome in children with associated proximal tubular injury

**DOI:** 10.3389/fneph.2024.1374200

**Published:** 2024-02-26

**Authors:** Silvia Maria Orsi, Carlotta Pepino, Lisa Rossoni, Margherita Serafino, Roberta Caorsi, Stefano Volpi, Serena Palmeri, Alessandro Faragli, Francesca Lugani, Carolina Bigatti, Gian Marco Ghiggeri, Enrico Eugenio Verrina, Edoardo La Porta, Andrea Angeletti

**Affiliations:** ^1^ Department of Neuroscience, Rehabilitation, Ophthalmology, Genetics, Maternal and Child Health, University of Genoa, Genoa, Italy; ^2^ Department of Pediatric Cardiology and Cardiac Surgery, IRCCS Istituto Giannina Gaslini, Genoa, Italy; ^3^ Center for Autoinflammatory Diseases and Immunodeficiencies, IRCCS Istituto Giannina Gaslini, Genoa, Italy; ^4^ Department of Internal Medicine and Cardiology, Charité – Universitätsmedizin Berlin, Berlin, Germany; ^5^ Division of Nephrology, Dialysis and Transplantation, IRCCS Istituto Giannina Gaslini, Genoa, Italy; ^6^ Laboratory of Molecular Nephrology, IRCCS Istituto Giannina Gaslini, Genoa, Italy; ^7^ Dialysis Unit, Department of Pediatric, IRCCS Istituto Giannina Gaslini, Genoa, Italy

**Keywords:** MIS-C multisystem inflammatory syndrome in children, proximal tubule injury, bladder debris, Fanconi syndrome, kidney injury, SARS – CoV – 2

In the published article, there was an error regarding the affiliation for Serena Palmeri. As well as having affiliation 3, they should also have affiliation *1, Department of Neuroscience, Rehabilitation, Ophthalmology, Genetics, Maternal and Child Health, University of Genoa, Genoa, Italy*.

In addition, in the published article an author name was incorrectly written as “Serena Palmieri”. The correct spelling is “Serena Palmeri”.

The authors apologize for these errors and state that they do not change the scientific conclusions of the article in any way. The original article has been updated.

